# Coastal development and precipitation drive pathogen flow from land to sea: evidence from a *Toxoplasma gondii* and felid host system

**DOI:** 10.1038/srep29252

**Published:** 2016-07-26

**Authors:** Elizabeth VanWormer, Tim E Carpenter, Purnendu Singh, Karen Shapiro, Wesley W. Wallender, Patricia A. Conrad, John L. Largier, Marco P. Maneta, Jonna A. K. Mazet

**Affiliations:** 1 One Health Institute, School of Veterinary Medicine, University of California, Davis, 1089 Veterinary Medicine Drive, Davis, CA, 95616, USA; 2 EpiCentre, Massey University, Private Bag 11222, Palmerston North 4442, New Zealand; 3 Department of Civil Engineering, VNR Vignana Jyothi Institute of Engineering and Technology, Bachupally Nizampet (S.O), Hyderabad-500090, India; 4 Department of Land, Air, and Water Resources, University of California, Davis, 1 Shields Ave, Davis, CA, 95616, USA; 5 Department of Pathology, Microbiology and Immunology, School of Veterinary Medicine, University of California, Davis, 1 Shields Ave, Davis, CA, 95616, USA; 6 Department of Environmental Science and Policy, University of California, Davis, Bodega Marine Laboratory, 2099 Westside Rd, Bodega Bay, CA, 94923, USA; 7 Department of Geosciences, University of Montana, 32 Campus Dr. #1296, Missoula, MT, 59812, USA.

## Abstract

Rapidly developing coastal regions face consequences of land use and climate change including flooding and increased sediment, nutrient, and chemical runoff, but these forces may also enhance pathogen runoff, which threatens human, animal, and ecosystem health. Using the zoonotic parasite *Toxoplasma gondii* in California, USA as a model for coastal pathogen pollution, we examine the spatial distribution of parasite runoff and the impacts of precipitation and development on projected pathogen delivery to the ocean. Oocysts, the extremely hardy free-living environmental stage of *T. gondii* shed in faeces of domestic and wild felids, are carried to the ocean by freshwater runoff. Linking spatial pathogen loading and transport models, we show that watersheds with the highest levels of oocyst runoff align closely with regions of increased sentinel marine mammal *T. gondii* infection. These watersheds are characterized by higher levels of coastal development and larger domestic cat populations. Increases in coastal development and precipitation independently raised oocyst delivery to the ocean (average increases of 44% and 79%, respectively), but dramatically increased parasite runoff when combined (175% average increase). Anthropogenic changes in landscapes and climate can accelerate runoff of diverse pathogens from terrestrial to aquatic environments, influencing transmission to people, domestic animals, and wildlife.

Forty percent of the world’s population lives in coastal areas^1^, where parasites, viruses, and bacteria flow to the ocean from terrestrial sources (e.g. faeces) through streams and rivers, storm drains, and overland freshwater runoff 2,3. Like sediment, nutrient, and chemical runoff, this terrestrially derived pathogen pollution can impact health in coastal systems^4^,^5^,^6^,^7^,^8^. Understanding how climate variability and human influences on land use shape land-sea pathogen flow is critical^3^,^8^, but these factors are often challenging to evaluate in natural settings. California offers a living laboratory for examining the effects of development and precipitation on coastal pathogen pollution. From 1910–2010, California’s human population, the majority of which resides in coastal counties, expanded from 2.4 to over 37 million, with close to 50 million expected by 2050^9^,^10^. Human population growth also increased domestic animal numbers; converted natural landscapes to commercial, residential, and agricultural uses; and added impervious surfaces that accelerate overland flow of contaminated runoff. These modifications have the potential, individually and in combination, to enhance land-sea pathogen flow. We use the term coastal development to collectively describe human population growth and associated increases in domestic animal numbers and developed lands in watersheds bordering the ocean. Climate change may also facilitate pathogen delivery to the ocean^3^. California projections suggest that even a decrease in overall rainfall may be accompanied by an increase in the intensity of individual storm events, which can increase contaminated runoff^11^.

*Toxoplasma gondii* is a globally distributed, terrestrial parasite that impacts public and animal health^12^. In California, high levels of *T. gondii-*related mortality in the threatened southern sea otter (*Enhydra lutris nereis*) population and human susceptibility to this parasite triggered concern about emerging coastal pathogen pollution^13^. As no marine hosts are known to shed *T. gondii*, infections in diverse marine mammals around the world suggest that land-sea transmission is common. *Toxoplasma gondii* infects warm-blooded animals, and exposure typically occurs through eating tissues of an infected host; congenital transmission; or ingesting free-living oocysts in contaminated water, soil, or food^12^,^14^. Environmental oocyst-based transmission, including waterborne outbreaks in humans, is increasingly recognized as an important source of infection^15^. Domestic and wild felids, the only known definitive hosts of the parasite, can shed hundreds of millions of oocysts following infection^16^,^17^, which can be transported to the ocean in freshwater runoff^13^,^18^. The exceptionally hardy oocysts can remain viable for over a year in soil, freshwater, and saltwater^19^,^20^, and the infectious dose may be as low as one oocyst^14^. *Toxoplasma gondii* exposure in otters may occur by ingesting oocysts in seawater or, more likely, through consumption of contaminated invertebrate prey^13^,^21^, including raw seafood that is also coveted by people^22^. Human infection with *T. gondii* can be asymptomatic. However, severe disease and death in people with compromised immune systems and in healthy individuals exposed to certain *T. gondii* strains underscore public health concerns of land-sea pathogen flow^12^,^23^.

Building upon empirical data collected through laboratory and field experiments^13^,^24^,^25^,^26^,^27^, we simulated *T. gondii* oocyst delivery from felid faeces to the ocean for watersheds along the California sea otter range (see Methods). We linked spatial domestic and wild felid oocyst loading models with a wet season hydrology model to evaluate the spatial distribution of coastal oocyst loading and the impacts of precipitation and coastal development on the parasite load reaching nearshore waters. Native wild felids included mountain lions (*Puma concolor*) and bobcats (*Lynx rufus*). Non-native domestic cats (*Felis catus*) included pet cats that defecate outdoors and free-ranging, unowned or “feral” domestic cats. Watershed landscapes varied from predominantly natural habitat with few human inhabitants to populated rural and urban areas with agricultural, residential, industrial, and commercial development. Based on soil infiltration rates typical of these watersheds, we used a precipitation intensity threshold capable of generating overland runoff and mobilizing pathogens from faeces in the hydrology model (4mm/hr; see Methods). At this threshold, contaminated runoff rapidly reaches the ocean from lands bordering the coast (Fig. 1). Using the coupled *T. gondii* oocyst loading and transport models, we provide evidence that levels of oocyst runoff from coastal watersheds and relative contributions of domestic and wild felids to oocyst runoff vary spatially along the sea otter range, and that changes in both development and precipitation can greatly enhance land-to-sea oocyst flow.

## Results and Discussion

We found a greater than 300-fold difference in total projected oocyst delivery from land to sea among the watersheds (Fig. 2). In watersheds of similar size, larger parasite loads were found in those with high human development compared to watersheds draining less disturbed areas. Both domestic and wild felids have the potential to shed *T. gondii* oocysts that can be carried by freshwater runoff to the ocean where sea otters are exposed. Although *T. gondii* delivery along portions of the coast is dominated by oocysts from wild felids, the large domestic cat contributions in developed areas highlight the potential for introduced, domestic animals to play an important role in land-sea pathogen flow.

Field studies, genetic evidence, and recent marine mammal mortality reports support the importance of domestic cat contributions to oocyst delivery to the ocean. Prevalence of *T. gondii* infection was higher in wild felids sampled in central coastal California and southern California^24^,^28^, but domestic cats likely contribute a much higher number of oocysts to coastal environments due to their larger population sizes^24^. Both domestic cats and wild felids sampled near the sea otter range were infected with the genotypes of *T. gondii* commonly identified in infected otters and contaminated prey species^29^,^30^,^31^. The prevalence of infection with Type X, the genotype identified in the majority of *T. gondii*-infected otters^13^,^32^, was higher in mountain lions and bobcats than domestic cats. However, increased coastal development has the potential to drive overlap between domestic and sylvatic cycles of *T. gondii* transmission, resulting in more Type X and atypical genotype infections in domestic cats^29^. Mortality due to *T. gondii* in marine mammals living near coastal landscapes without wild felids (Monk seals (*Monachus schauinslandi*) near Hawaii^33^ and Hector’s dolphins (*Cephalorhyncus hectori*) endemic to New Zealand^34^) provides additional evidence of the potential for domestic cats to play an important role in marine oocyst loading.

In the absence of standardized methods that can efficiently detect *T. gondii* oocysts in large volumes of water, we cannot directly measure oocyst delivery to the ocean. However, spatial patterns of sea otter infection offer a proxy for the oocyst load in coastal waters. Watersheds with the highest estimated total and domestic felid parasite delivery to the ocean align closely with areas of moderate to high levels of *T. gondii* infection in live and dead sea otters^13^,^21^ (Fig. 2). While the alignment is not perfect, live sea otter movement patterns, drift of sampled sea otter carcasses, prey preferences of sampled sea otters^21^, and redistribution of oocysts in the ocean due to physical and ecological processes may account for some of the observed spatial discrepancies.

Chemical and physical properties of oocysts coupled with ecological processes in the near-shore marine environment can influence oocyst transport and sea otter exposure. The hydrophilic nature of oocysts and their negative surface charge in freshwater reduces likelihood of attachment to other particles or vegetation, thus enhancing their potential to be mobilized from faeces and efficiently transported in surface waterways^26^. In saltwater, oocyst surface charge becomes neutral, making them more likely to adhere to other particles or vegetation and settle out of the water column, especially in estuaries and near-shore marine environments where fresh and salt waters mix^26^. As sea otters commonly spend time in these environments, oocyst surface properties may play a large role in determining high-risk zones of *T. gondii* accumulation, marine invertebrate oocyst retention, and sea otter infection. In the nearshore marine environment, oocysts can be incorporated in macroaggregates of particles (i.e., “marine snow”) and sink to the ocean floor or be captured by sticky kelp surfaces where invertebrates and sea otters can be exposed^35^. Recent evidence suggests that aquatic polymers, such as those found on kelp, shape marine patterns of sea otter pathogen exposure^36^, highlighting the importance of linking terrestrial and aquatic pathogen loading and transmission processes to understand marine health risks. Future modeling efforts to incorporate marine oocyst transport, particle dynamics, and sea otter ecology will complement our current estimates of relative oocyst runoff from terrestrial watersheds to the ocean.

Basing our modelling scenarios on realistic felid and environmental input values (see Methods), we show that increasing coastal development and precipitation can greatly increase oocyst delivery to the ocean. Coastal development and precipitation conditions were simulated in a subset of 5 “indicator” watersheds (Fig. 3). We selected watersheds with a mixture of developed and undeveloped land-use types; habitats suitable for domestic and wild felid populations; and hourly rainfall data available from a precipitation gauge within or near the given watershed (Supplementary Figs S1 and S2). To reflect actual California coastal development (increases in domestic cat numbers and developed lands accompanying human population growth) from 1990 to 2010, model inputs were drawn from historical spatial data (household census and land cover data; Supplementary Table S2). In the indicator watersheds, the number of households increased 40–60%, estimated domestic cat numbers rose 25–50%, and developed land area grew 80–150% over the 20-year period. To simulate realistic inter-annual variability in precipitation, we incorporated historic hourly rainfall data for each watershed for low and high precipitation wet seasons (see Methods; Supplementary Fig. S2).

We compared relative trends of pathogen delivery from the indicator watersheds under four scenarios that incorporated different levels of development and precipitation. Model scenarios included: 1) baseline development (1990 households and land use) with low precipitation; 2) increased coastal development (2010 households and land use) with low precipitation; 3) baseline development with high precipitation; and 4) increased development and high precipitation (Fig. 3). For all watersheds, the historically observed increase in coastal development or precipitation alone increased oocyst delivery to the ocean (average increases of 44% and 79%, respectively). However, combined increases in coastal development and precipitation synergistically increased oocyst delivery (average 175% increase). With 100 iterations of each scenario, the trend of greatly increased oocyst delivery with increased coastal development and observed high precipitation levels is consistent across watersheds (Fig. 3).

The increase in *T. gondii* oocyst delivery due to coastal development highlights the role of human landscape and animal management in disease emergence and transmission^37^. California settlers built cities along rivers and the coast to take advantage of resource and transport opportunities. Continued human population growth at these sites increases domestic animal numbers and fecal loading in areas where pathogen transport in runoff is enhanced by impervious surfaces near natural and channelized waterways, storm drains, or the ocean. Loss and reduction of wetlands, which filter pathogens like *T. gondii* from runoff 25, further exacerbates the impacts of coastal development. Almost two-thirds of US saltwater marshes have been degraded^38^, and 90% of California’s wetlands have been lost^39^. Landscape conversion and rising sea levels may further reduce coastal wetlands^39^ and enhance transport of terrestrially- derived pathogens to the ocean. Climate-change impacts on precipitation are difficult to predict, but modelled oocyst delivery trends suggest that even observed levels of inter-annual variability in California precipitation may greatly increase pathogen load reaching the ocean. Management actions that decrease domestic animal defecation in coastal watersheds; reduce impervious surfaces; and maintain or restore coastal and riparian wetlands could mitigate the impact of human population growth, land use change, and climate variability on land-sea pathogen flow. Decreased runoff of terrestrial pathogens is a previously unrecognized human and animal health benefit of proposed ecosystem-based coastal defence strategies, including wetland creation and restoration, designed to reduce coastal flooding risk^6^.

Using field and laboratory data to inform our assumptions on pathogen shedding, survival, and transport, we aimed to estimate relative levels of oocyst delivery to the ocean rather than absolute numbers. Incorporation of historic California development and precipitation data allowed us to evaluate realistic trends in oocyst delivery. Our results demonstrate the importance of human influences in shaping the spatial pattern of pollution with a model zoonotic pathogen*, T. gondii*, in coastal waters of California and enhancing pathogen flow from land to sea. As human and associated domestic animal populations increase globally, policies and management actions to reduce coastal pathogen pollution will play a critical role in protecting health at the human-animal-environment interface.

## Methods

Linked oocyst loading and hydrologic transport models were created in ArcGIS version 10.2 (Environmental Systems Research Institute, Redlands, California, USA). Given differences in felid demography, terrestrial distribution, behaviour, and oocyst shedding, we constructed four separate stochastic models for outdoor pet cats, unowned feral domestic cats, mountain lions, and bobcats to simulate relative oocyst loading in coastal watersheds. Free-ranging, unowned or “feral” domestic cat populations can be further divided as “managed” and “unmanaged”, with managed feral cats dependent upon humans for food or shelter resources and unmanaged feral cats surviving independently of humans on predominantly prey-based diets^24^. The distribution of pet and managed feral cat populations is closely linked to human households in developed landscapes^40^. Unmanaged, more solitary feral cats not fed or cared for by humans may inhabit urban and agricultural lands as well as less developed areas^40^. Given the uncertainty in estimating density and shedding prevalence for unmanaged feral cats due to limited reports and small sample sizes in California field studies, these felids were excluded from the feral domestic cat loading model. Exclusion from the model is not anticipated to strongly influence projected domestic cat oocyst delivery to the ocean as coastal unmanaged feral cat populations are estimated to be small relative to the populations of pet cats and managed feral cats^24^. If included, unmanaged feral cats would increase the number of domestic cat source oocysts estimated to flow to the ocean.

Domestic and wild felid loading models were linked with a modified deterministic rainfall-runoff hydrology model (details below). Oocyst loading was estimated for one year, and October 1 through May 1 wet season precipitation levels for a given water year were used in the transport model. Publicly available GIS data layers were used to define the study area, provide input data, and evaluate model scenarios (Supplementary Table S1). Stochastic and deterministic model parameters were derived from literature values and California field data (Supplementary Table S2). Oocyst transport in overland and channel freshwater flow was simulated based on precipitation data recorded at multiple gauges in the study area.

### Defining the study area

**Figure 1:**
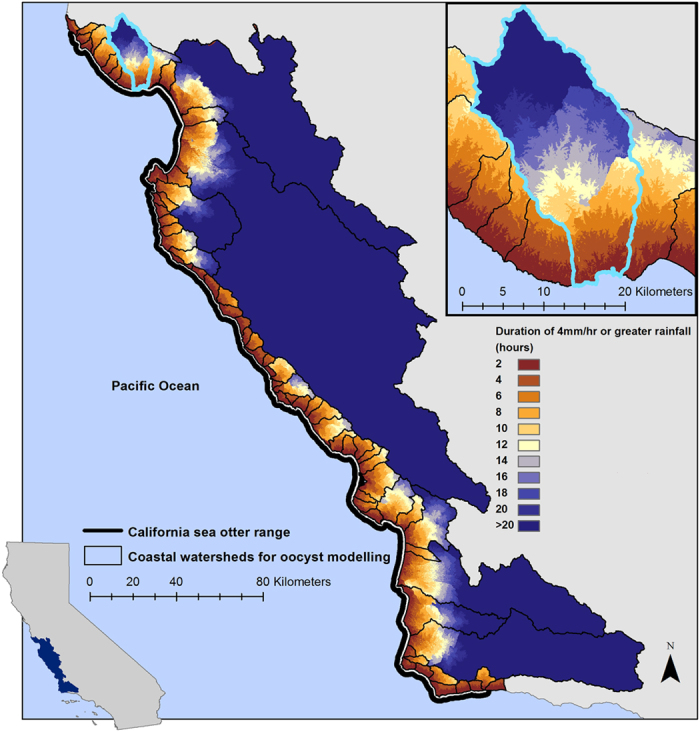
Cumulative transport-time required for *Toxoplasma gondii* oocyst-contaminated runoff to reach the ocean from watersheds bordering the California sea otter range. Transport-time estimates (red to blue shading) represent hours of 4mm/hr or greater intensity rainfall. The larger scale inset illustrates transport-time for the San Lorenzo River watershed (outlined in light blue). Transport patterns were simulated using 2010 coastal land use data, and the map was created using ArcGIS software version 10.2 (ESRI; http://www.esri.com/software/arcgis/arcgis-for-desktop). For details on simulation of pathogen transport, see Methods.

The linear range of the sea otter population along the California coast^41^ was intersected with a hydrological stream network layer^42^, and 71 watersheds of the streams/rivers flowing into the Pacific Ocean formed the study area in which oocyst loading and transport models were implemented (Fig. 1; Supplementary Table S1). The boundaries of each watershed included all CalWater 2.2.1^43^ planning watershed units of an identified stream/river and its tributaries. These watersheds cover approximately 22,000 km^2^ and vary in size (~13 to 9,000 km^2^), human population density, land use, and freshwater runoff patterns. For all loading and transport models, the study area was ultimately converted to a raster of 30 × 30 m grid cells, consistent with the publicly available elevation data raster (Supplementary Table S1).

### Modelling oocyst loading

Total oocyst loading for a given 30 × 30 m grid cell was estimated for one year by summing simulated oocyst numbers contributed by all felids (domestic and/or wild) potentially defecating in that cell. Land-use layers (Supplementary Table S1) delineated urban, agricultural, grassland, scrub, wetland, and forest habitat, which in turn provided information on landscape-scale distribution of developed and wild land locations^44^,^45^,^46^. Domestic cat shedding was concentrated around human households, whereas wild felid shedding was distributed in less developed areas. Household locations within watersheds were modeled on the aggregate level of US census blocks^47^, which offer the most detailed spatial household data publicly available for the entire study area. The distribution of households within census blocks was determined according to land use. In census blocks containing only urban or rural developed lands (agricultural, residential, industrial or commercial land uses), households were assumed to be uniformly distributed across the landscape, whereas in census blocks with both developed and undeveloped lands, households were allocated to the developed areas. Developed lands containing one or more households were buffered by average pet cat or feral cat home ranges (120 m or 170 m, respectively^40^,^48^,^49^) to define zones of domestic cat shedding related to cat movement in the landscape. Following the creation of buffers, the developed area (km^2^) in each census block was recalculated, and the number of households was divided by the number of 30 × 30 m grid cells in that area to generate an estimate of households per grid cell. In loading models for pet cats and feral domestic cats, total households were multiplied by a discrete pet or feral cat per household value (Supplementary Table S2) to obtain an estimate of the domestic cat numbers in a given grid cell. Pet and feral cat per household parameter values were based on surveys conducted in central coastal California^50^. Census blocks with no households, containing only state management lands or protected areas, or containing only water or wetlands were assigned zero values for oocysts shed by domestic felids. Wild felid populations were distributed in undeveloped areas of coastal watersheds (i.e. lands not used for agriculture, residential, industrial, or commercial purposes) in the loading model. Total numbers of wild felids in a grid cell were estimated from stochastic distributions of felid density (Supplementary Table S2) based on reported densities of bobcats or mountain lions per km^2^.

For each felid group, grid cell oocyst load was calculated as the product of total number of animals in the cell, oocyst shedding prevalence, and oocysts shed per felid shedding event. Oocyst shedding prevalences were based on published literature values for outdoor pet cats in California and recent field data for feral cats and wild felids (Supplementary Table S2). Reported numbers of oocysts shed per individual following *T. gondii* infection range from zero to hundreds of millions of oocysts in experimental studies^16^,^17^. Given the extreme diversity in reported values and limited data on natural shedding in domestic and wild felids, a standard value of 50 million oocysts was chosen as a representative estimate for an average shedding event. This value has been used to estimate environmental oocyst burden in diverse geographic sites^27^,^51^.

The oocyst-loading model for each felid group incorporated stochastic parameters, such as felid density and/or oocyst shedding prevalence (Supplementary Table S2). To address the variability and uncertainty in wild felid density reports, statistical distributions for mountain lion and bobcat density were fitted to published values for each group using @RISK v. 5.0 (Palisade Corporation, Ithaca, New York). Distribution fit was evaluated by Chi-square, Kolmogorov-Smirnov, and Anderson-Darling statistical tests, and the loglogistic distributions included in the loading model fit the data well in all three tests (Supplementary Table S2). Beta distributions, which have commonly been applied to proportion estimates, were used to model the field- and literature-derived oocyst shedding prevalences. Outdoor pet cat and feral domestic cat oocyst loading estimates generated for each cell in the model were combined to produce a “domestic felid” loading raster. Mountain lion and bobcat oocyst loading estimates for each cell were combined to produce a “wild felid” loading raster. General loading model sensitivity analyses were performed in @RISK to evaluate the influence of all input parameters shown in Supplementary Table S2 on the outcome of total oocyst load. The loading model was most sensitive to oocyst shedding prevalence parameters. In light of this finding, a strength of the model lies in the use of local oocyst shedding prevalence estimates taken from field studies of wild and domestic felids living in the modeled watersheds^24^.

### Modelling oocyst transport

Oocyst transport in overland and channel flow was estimated using a parsimonious rainfall-runoff hydrologic modelling approach based on a time-area routing technique^52^. This technique uses precipitation intensities above the runoff production threshold and terrain properties to determine the progressive increase in area within a catchment contributing flow into the ocean at set time increments. This approach can be parameterized with published data and has shown good performance for coastal regions^53^. Velocities of overland and channel freshwater flow were used to determine the duration of threshold intensity precipitation (≥4 mm/hr) required for rain falling on a particular location in a watershed to reach the outflow point (Fig. 1), whether rain fell continuously or in several events. In the case of *T. gondii* transport, it was the cumulative hours of threshold intensity rainfall (≥4 mm/hr) during a wet season required for an oocyst entrained in freshwater flow to move from a given cell in a watershed to the ocean.

To create the hydrologic transport model, a national digital elevation model (DEM) with cell resolution of 30m was clipped to the study area boundaries. Using the hydrology functions in the ArcGIS v.10.2 Spatial Analyst toolbox, the DEM was filled and used to calculate slope fraction, flow direction, flow accumulation, and distance along the flow plane. Flow accumulation values were used to classify surface flow into overland and channel flow. Overland flow velocity (V_o_) was calculated following an Equation given by Dervos *et al.*^52^


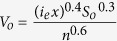


where *i*_*e*_ represents excess rainfall intensity (in m/s), *x* represents distance along the flow plane (m), *S*_*o*_ represents decimal slope, and *n* is the Manning’s roughness coefficient for vegetation.

The roughness coefficient affects the estimated speed of freshwater flow over specific land covers such as forest, grassland, and impervious surfaces. The roughness coefficient raster was derived from land use maps for the study area^45^,^46^, and grid cell values were assigned using published Manning’s coefficient estimates for specific land uses^52^,^54^,^55^. Transport models for 1990 and 2010 coastal development scenarios used different roughness coefficient rasters based on the distribution of land use. Using the calculated overland flow velocity and average channel flow velocity, transport time from each watershed grid cell to the outflow point was estimated using the Spatial Analyst *flowlength* function.

Oocysts are transported by overland flow that occurs when rainfall intensity exceeds the infiltration rate of the soil, which is influenced by soil type, vegetation cover, and soil saturation level. The 4 mm/hr precipitation intensity used as a threshold for generating overland flow was based upon soil infiltration rates typical of the modelled watersheds^56^. Additional factors influence oocyst transport in overland runoff from a given location, including slope of the land, vegetation, velocity of freshwater flow, age of faeces, and felid behaviour - whether faeces are buried or left exposed (as reviewed in^57^). Duration of threshold intensity rainfall (measured in hours) from October 1 through May 1 was determined for the 1991 and 2011 water years using hourly rainfall intensity data at California Irrigation Management Information System (CIMIS) gauge stations^58^ along the sea otter range (Supplementary Fig. S2). The US water year runs from October 1 through September 30, but precipitation data for the models were restricted to October 1 through May 1 to reflect the typical California wet season. Thus, the transport models combined duration of all 4 mm/hr or greater intensity precipitation events for an entire wet season. The cumulative time-travel isochrone corresponding to the duration of ≥4 mm/hr rainfall for a given scenario was multiplied by the oocyst loading grids to determine number of oocysts from each felid group transported to ocean. Oocyst totals from grid cells in the appropriate isochrone were summed for each watershed using zonal statistics in the Spatial Analyst toolbox and multiplied by a mobilization coefficient (percent of oocysts mobilized from faeces).

Although precipitation intensity impacts the pathogen mobilization from faeces, the percentage of *T. gondii* oocysts mobilized under different intensities of rainfall is unknown. We used a conservative estimate of 1% mobilization for domestic and wild felids based on experimental and field studies of the related parasite, *Cryptosporidium parvum.* Those studies, conducted at higher intensities of rainfall (25–64 mm/hr), estimated that between 0.05 and 59 percent of oocysts were mobilized from fresh faeces^59^,^60^; the proportion decreased in drier, “aged” faeces^60^. Under natural rainfall and rangeland conditions in California, storm events of 4–8 mm/hr rainfall intensity were able to mobilize *Cryptosporidium* oocysts and transport them in overland freshwater flow, though the percentage was not estimated^61^. *Toxoplasma gondii* oocyst load in coastal watersheds was estimated for one year before the hydrology model was run to create an initial distribution of parasites in the environment for transport in freshwater runoff during wet season storms. Given the exceptionally hardy nature of *T. gondii* oocysts and documented survival greater than one year in soil, freshwater, and seawater in temperate climates, an oocyst decay term was not used in the loading or transport models.

### Modelling the spatial distribution of oocyst loading along the sea otter range

**Figure 2:**
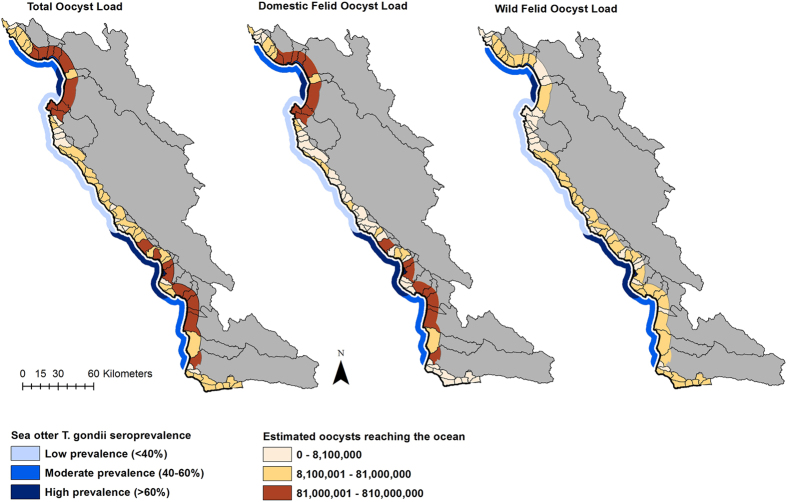
Spatial distribution of *Toxoplasma gondii* oocysts carried to the ocean via freshwater runoff (light yellow to red shading). The blue shading illustrates sea otter *T. gondii* seroprevalence from published field studies. Oocyst delivery patterns were simulated using linked GIS-based *T. gondii* oocyst loading and transport models (see additional detail in Methods) with 2010 California coastal land-use and human population data and 2011 water year precipitation data. All maps were created using ArcGIS software version 10.2 (ESRI; http://www.esri.com/software/arcgis/arcgis-for-desktop).

For each felid type (domestic or wild) and each coastal development scenario (baseline 1990 values or increased 2010 values), the loading model was run 100 times to generate 100 unique loading rasters using iterative loading model code developed in Python v. 2.6. To examine the spatial distribution of loading along the sea otter range (as seen in Fig. 2), the 100 wild and 100 domestic felid loading rasters for the increased development census and land-use data were averaged using the weighted sum function to produce a mean final loading layer for each felid type. This mean oocyst loading layer provided input data for the transport model to create a mean oocyst delivery raster under 2011 water year precipitation conditions (Supplementary Fig. S2).

### Modelling coastal development and precipitation scenarios

**Figure 3:**
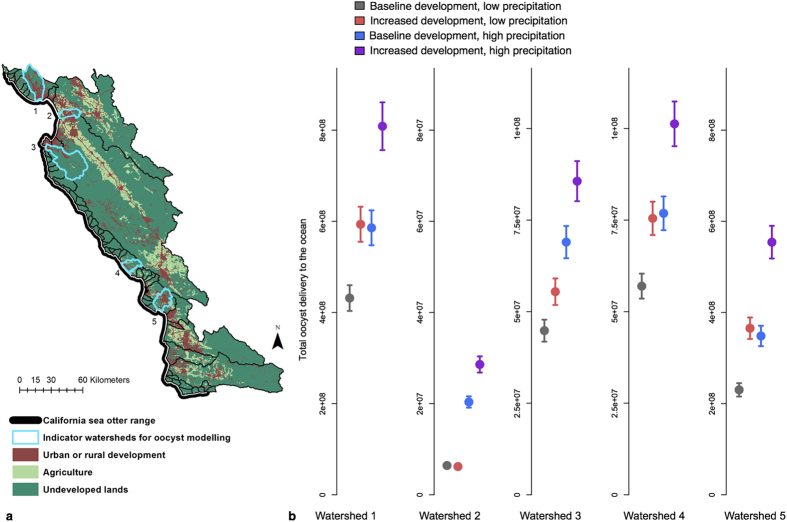
Pathogen delivery to the ocean under varied coastal development and precipitation conditions. (**a)** *Toxoplasma gondii* oocyst delivery was simulated for 5 indicator watersheds (highlighted in blue and numbered from north to south). The map was created using ArcGIS software version 10.2 (ESRI; http://www.esri.com/software/arcgis/arcgis-for-desktop). (**b)** Mean oocyst delivery by watershed for 100 iterations of 4 development and precipitation scenarios. Error bars show 95% confidence intervals. Scenarios evaluated included 1) baseline development (1990 households/land use) with low precipitation (grey); 2) increased coastal development (2010 households/land use) with low precipitation (red); 3) baseline development with high precipitation (blue); and 4) increased development and high precipitation (purple).

Scenarios were modelled in indicator watersheds with heterogeneous land use, habitat suitable for domestic and wild felid populations, and hourly rainfall data available for 1991 and/or 2011 water years from a CIMIS precipitation gauge within or near the given watershed (Supplementary Figs S1 and S2). For 1990 and 2010 coastal development conditions, human household numbers and area (km^2^) of developed urban or rural land use (commercial, industrial, residential, and agricultural) from human census and land cover layers, respectively, were summed within each watershed. The percent change in households and developed land area over the 20-year period was used to select the subset of 5 indicator watersheds with both household and developed land-use growth. “Low” precipitation scenario values for each watershed were hours of threshold intensity precipitation from October 1 through May 1 in the 1991 water year (Supplementary Fig. S2). “High” precipitation scenarios used hours of threshold intensity precipitation from October 1 through May 1 in the 2011 water year. To evaluate the coastal development and precipitation scenarios in the five indicator watersheds, each of the 100 unique loading rasters was used as an input to the transport model to create 100 oocyst delivery rasters for each scenario. In each indicator watershed, mean numbers of oocysts with 95% confidence intervals for the four scenarios illustrate differences in projected oocyst delivery to the ocean (Fig. 3).

## Additional Information

**How to cite this article**: VanWormer, E. *et al.* Coastal development and precipitation drive pathogen flow from land to sea: evidence from a *Toxoplasma gondii* and felid host system. *Sci. Rep.* **6**, 29252; doi: 10.1038/srep29252 (2016).

## Supplementary Material

Supplementary Information
