# Supplementary material for: Coastal development and precipitation drive pathogen flow from land to sea: evidence from a Toxoplasma gondii and felid host system
Source: Sci Rep. 2016 Jul 26;6:29252. doi: 10.1038/srep29252 (PMC4960600; doi:10.1038/srep29252)
Supplement: Supplementary Information [file srep29252-s1.pdf]

Coastal development and precipitation drive pathogen flow from land to sea: evidence from a *Toxoplasma gondii* and felid host system

Elizabeth VanWormer[1,*], Tim E Carpenter[2], Purnendu Singh[3,4], Karen Shapiro[1,5], Wesley W. Wallender[4], Patricia A. Conrad[1,5], John L. Largier[6], Marco P. Maneta[7], and Jonna A. K. Mazet[1,*]

[1]One Health Institute, School of Veterinary Medicine, University of California, Davis, 1089 Veterinary Medicine Drive, Davis, CA, USA, 95616

[2]EpiCentre, Massey University, Private Bag 11222, Palmerston North 4442, New Zealand

[3]Department of Civil Engineering, VNR Vignana Jyothi Institute of Engineering and Technology, Bachupally Nizampet (S.O) Hyderabad-500090, India

[4] Department of Land, Air, and Water Resources, University of California, Davis, 1 Shields Ave, Davis, CA, USA, 95616

[5]Department of Pathology, Microbiology and Immunology, School of Veterinary Medicine, University of California, Davis, 1 Shields Ave, Davis, CA, USA, 95616

[6]Department of Environmental Science and Policy, University of California, Davis, Bodega Marine Laboratory, 2099 Westside Rd, Bodega Bay, CA, 94923

[7]Department of Geosciences, University of Montana, 32 Campus Dr. #1296, Missoula, MT, 59812

* Corresponding Authors: Elizabeth VanWormer, evanwormer@ucdavis.edu; Jonna A. K. Mazet, jkmazet@ucdavis.edu

Supplementary Information

Supplementary Tables:

Table S1. Publicly available GIS data used to construct *Toxoplasma gondii* oocyst loading and transport models.

GIS Data Set	Data Source	Year of data collection (Y) or latest update (U)	Modeling Use	References
Sea otter range	USGS: Western Ecological Resource Center	2010 (Y)	Define study area	41
Stream network	California Department of Fish and Wildlife	2003 (U)	Define study area	42
Coastal watersheds	California Interagency Watershed Map: CalWater 2.2.1	2004 (U)	Define study area	43
Elevation	USGS: National Elevation Dataset (NED)	2010 (U)	Transport model; define slope, flow direction, flow accumulation and flow length	62
Land use				
California -specific	California Department of Conservation: Farmland Monitoring and Mapping Program	2010 (Y), 1990 (Y)	Define urban and agricultural areas	44
National (USA)	USGS: National Land Cover Dataset (NLCD)	2011 (Y), 1992 (Y)	Define developed and undeveloped land uses	45, 46, 63, 64
Census	US Census Bureau, Fire and Resource Assessment Program: Migrated Tiger Census Data	2010 (Y), 1990 (Y)	Define households in watersheds	47

Table S2. *Toxoplasma gondii* oocyst loading model felid demography and shedding parameters.

**Model parameter**	**Parameter Type**	**Distribution or Value**	**Data Source**
Mountain lion density	Stochastic	Loglogistic (0, 0.011173, 2.02)*	65, 66, Reviewed by 67
Bobcat density	Stochastic	Loglogistic (0, 0.31707, 3.5474)*	68-74
Managed feral cats per household	Deterministic	0.32 cats/household	50
Outdoor pet cats per household	Deterministic	0.21 cats/household	50
Oocyst shedding prevalences:			
Mountain lion	Stochastic	Beta (3, 50)	24
Bobcat	Stochastic	Beta (3, 15)	24
Managed feral cat	Stochastic	Beta (9, 428)	24
Pet cat	Stochastic	Beta (3, 152)	27
Oocysts shed per felid shedding event	Deterministic	50,000,000	27

* Distribution of data was not significantly different from the Loglogistic distribution in Chi-Square, Kolmogorov-Smirnov, or Anderson-Darling tests ($\alpha = 0.05$).

Supplementary Figures:

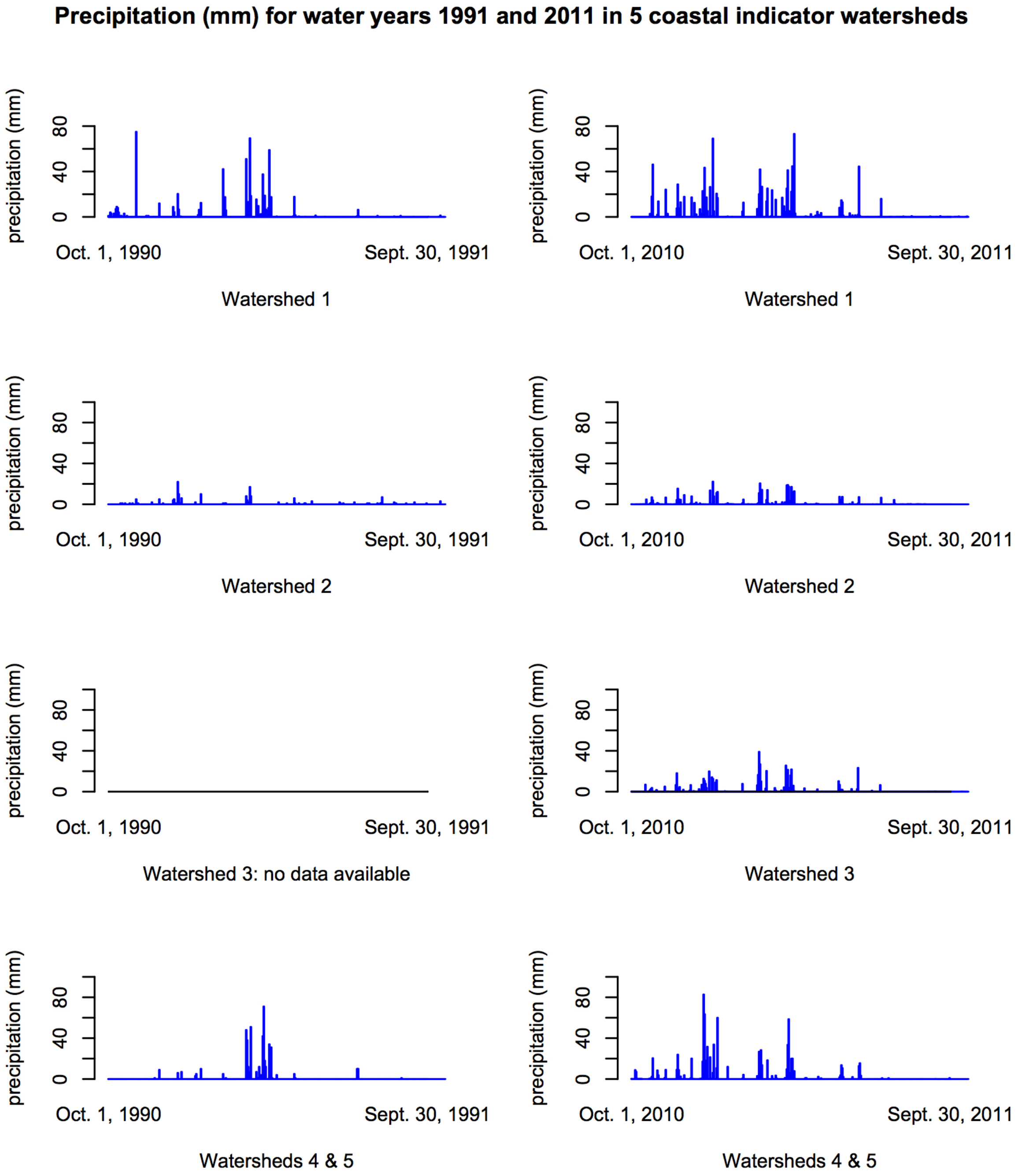

Figure S1. Total precipitation (in mm) for the 1991 and 2011 water years for the indicator watersheds (shown in Fig. 3) along the central California coast, USA.

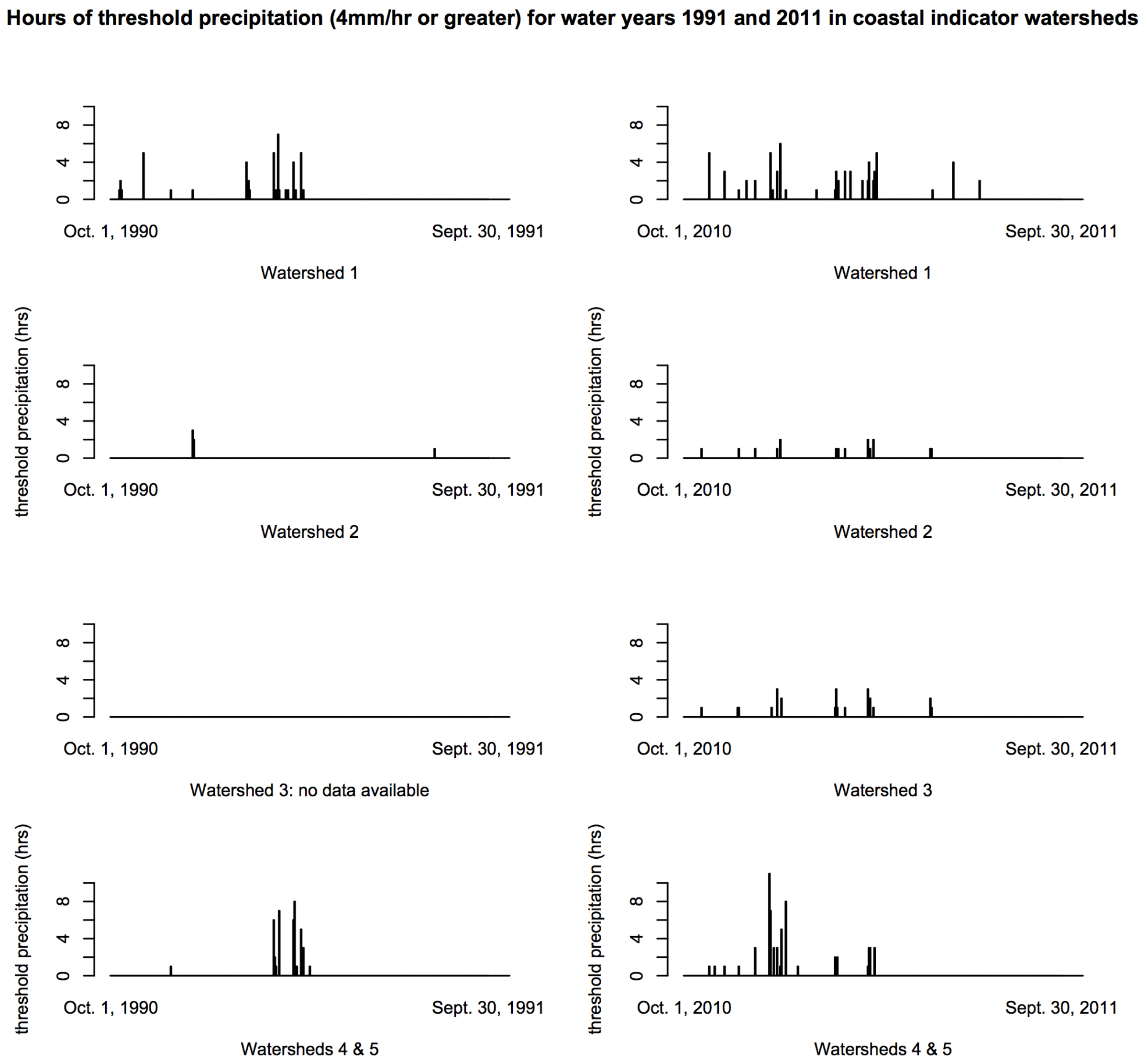

Figure S2. Duration (in hours) of ≥ 4mm/hr intensity precipitation for the 1991 and 2011 water years for the indicator watersheds (shown in Fig. 3) along the central California coast, USA.

Supplementary References:

62 United States Geological Survey. *National Elevation Dataset (NED).* (2010) Available at: http://atlas.ca.gov/ download.html#/casil/elevation/ned. (Accessed: 20th December 2011)

63 Gesch, D. *et al*. The national elevation dataset. *Photogramm. Eng. Rem. S.* **68**, 5-11 (2002).

64 Gesch, D. B. The national elevation dataset in *Digital elevation model technologies and applications: the DEM users manual* (ed. Maune, D.F.) 99-118 (American Society for Photogrammetry and Remote Sensing Publications, 2007).

65 Pierce, B. M., Bleich, V. C., Wehausen, J. D. & Bowyer, R. T. Migratory patterns of mountain lions: Implications for social regulation and conservation. *J. Mammal.* **80**, 986-992 (1999).

66 Laundré, J. & Clark, T. W. Managing puma hunting in the western United States: through a metapopulation approach. *Anim. Conserv.* **6**, 159-170 (2003).

67 Quigley, H. & Hornocker, M. Cougar population dynamics in *Cougar: ecology and conservation* (eds Hornocker, M. & Negri, S.) Ch 5, 59-75 (University of Chicago Press, 2009).

68 Larrucea, E. S., Serra, G., Jaeger, M. M. & Barrett, R. H. Censusing bobcats using remote cameras. *West. N. Am. Naturalist* **67**, 538-548 (2007).

69 Ruell, E. W., Riley, S. P. D., Douglas, M. R., Pollinger, J. P. & Crooks, K. R. Estimating bobcat population sizes and densities in a fragmented urban landscape using non-invasive capture-recapture sampling. *J. Mammal.* **90**, 129-135 (2009).

70 Heilbrun, R. D., Silvy, N. J., Peterson, M. J. & Tewes, M. E. Estimating bobcat abundance using automatically triggered cameras. *Wildl. Soc. B.* **34**, 69-73 (2006).

71 Roberts, N. M. & Crimmins, S. M. Bobcat population status and management in North America: Evidence of large-scale population increase. *J. Fish Wildl. Manage.* **1**, 169-174 (2010).

72 Lawhead, D. N. Bobcat (*Lynx rufus*) home range, density and habitat preference in south-central Arizona. *Southwest. Nat.* **29**, 105-113 (1984).

73 Knick, S. T. Ecology of bobcats relative to exploitation and a prey decline in southeastern Idaho. *Wildl. Monogr.* **108**, 1-42 (1990).

74 Riley, S. D. *et al*. Effects of urbanization and habitat fragmentation on bobcats and coyotes in southern California. *Conserv. Biol.* **17**, 566-576 (2003).
